# Isolation and characterization of polymorphic microsatellite loci for the three Iberian vipers, *Vipera aspis*, *V. latastei* and *V. seoanei* by Illumina MiSeq sequencing

**DOI:** 10.1007/s11033-024-09263-5

**Published:** 2024-02-09

**Authors:** Inês Freitas, Guillermo Velo-Antón, Susana Lopes, Antonio Muñoz-Merida, Fernando Martínez-Freiría

**Affiliations:** 1 Centro de Investigação em Biodiversidade e Recursos Genéticos, CIBIO, InBIO Laboratório Associado, Universidade do Porto, Campus de Vairão, Vairão, 4485-661 Portugal; 2 BIOPOLIS Program in Genomics, Biodiversity and Land Planning, CIBIO, Campus de Vairão, Vairão, 4485-661 Portugal; 3 Departamento de Biologia, Faculdade de Ciências, Universidade do Porto, Porto, 4099-002 Portugal; 4 Facultad de Biología, Edificio de Ciencias Experimentales, Universidad de Vigo, Bloque B, Planta 2, Laboratorio 39 (Grupo GEA), Vigo, E-36310 Spain

**Keywords:** Cross-species transferability, Microsatellites, Polymorphic, *Vipera*

## Abstract

**Background:**

European vipers (genus *Vipera*) are a well-studied taxonomic group, but the low resolution of nuclear sanger-sequenced regions has precluded thorough studies at systematic, ecological, evolutionary and conservation levels. In this study, we developed novel microsatellite markers for the three Iberian vipers, *Vipera aspis*, *V. latastei* and *V. seoanei*, and assessed their polymorphism in north-central Iberian populations.

**Methods and results:**

Genomic libraries were developed for each species using an Illumina Miseq sequencing approach. From the 70 primer pairs initially tested, 48 amplified reliably and were polymorphic within species. Cross-species transferability was achieved for 31 microsatellites loci in the three target species and four additional loci that were transferable to one species only. The 48 loci amplified in average seven alleles, and detected average expected and observed heterozygosities of 0.7 and 0.55, in the three genotyped populations/species (26 *V. aspis*, 20 *V. latastei* and 10 *V. seoanei*).

**Conclusions:**

Our study provides a selection of 48 polymorphic microsatellite markers that will contribute significantly to current knowledge on genetic diversity, gene flow, population structure, demographic dynamics, systematics, reproduction and heritability in these species, and potentially in other congeneric taxa.

**Supplementary Information:**

The online version contains supplementary material available at 10.1007/s11033-024-09263-5.

## Introduction

Despite the increasing affordability and high throughput data of Next Generation Sequencing (NGS) technologies, microsatellite markers remain valuable tools in evolutionary biology, ecology, and conservation. These highly polymorphic, co-dominant and noncoding markers offer fine-scale resolution of genetic variation within and among populations [1] and thus, they are still widely used in population genetic research (e.g. [2, 3]).

European vipers (fam. Viperidae, genus *Vipera*) constitute a clade of venomous snakes distributed across Eurasia and North Africa. With up to 25 species described and multiple studies on phylogenetics, phylogeography and ecology, current knowledge at both interspecific and intraspecific level is still limited by the lack of informative nuclear DNA data [4]. This shortage is particularly evident in the limited number of population genetic studies (but see [5–7]), crucial for the conservation of declining viper populations. Nuclear markers were developed for three species (*Vipera berus* [5, 8], *Vipera ursinii* [9], *V. aspis* [10]), but their high specificity limits a broader application across the group. For instance, *V. berus* markers were applied in a contact zone between the three Iberian vipers, *V. aspis*, *V. latastei* and *V. seoanei*, to detect hybridization [11]. Although they were able to cross-amplify in these species and provided enough resolution to identify hybrids and parentals, they failed to recover intraspecific structure due to low polymorphism (authors, unpublished data).

While 12 microsatellite loci have been already developed for *V. a. aspis* populations (from Switzerland [10]), the Iberian endemics *Vipera latastei* and *Vipera seoanei* lack suitable nuclear markers. *Vipera aspis* shows high genetic and phenotypic diversity and deep genetic divergence that granted the description of four subspecies [12]. *Vipera aspis* is listed as Least Concern but at least two of the subspecies face significative population decline and are listed as Critically Endangered (*V. a. aspis*) and Endangered (*V. a. francisciredi*) in regional assessments. Developing a new battery of markers for other subspecies can enhance resolution to address several aspects of evolutionary history, demography, reproduction and conservation that are still understudied. Similarly, mitochondrial DNA (mtDNA) studies recovered deep evolutionary lineages and high genetic diversity within *V. latastei* [13–15]. However, nuclear markers are needed to address reproductive isolation and the extent of gene flow among divergent lineages, aiding in clarifying their taxonomic status [15]. *Vipera seoanei*, on the other hand, exhibits a shallow genetic structure and low genetic diversity at the mtDNA level [16], that contrasts with its high morphological and colouration polymorphism [17, 18]. The use of microsatellite loci has the potential to provide a detailed perspective on the genetic diversity and structure of populations and the factors contributing to their phenotypic variation. *Vipera latastei* is listed as Vulnerable, due to significant population fragmentation and decline, and *V. seoanei* is listed as Least Concern, although southern populations are presumably isolated and highly vulnerable to climate change [19]. Yet, genetic studies assessing population diversity and gene flow are lacking for these species.

To address these gaps, we have developed a new set of polymorphic microsatellite markers specific for each of the three Iberian vipers. We tested these markers for polymorphism in north-central Iberian populations and explored cross-amplification across the three target species. With this initiative, we aim to contribute with molecular tools with which we can: (1) characterize patterns of genetic diversity and population structure at an intraspecific level; (2) investigate gene flow and reproductive isolation in contact zones between species and lineages; and (3) perform paternity tests for various ecological and evolutionary questions, ultimately informing effective conservation strategies for each species.

## Materials and methods

Genomic libraries were prepared using high molecular weight genomic DNA from 10 tissue samples of each species, collected across their distribution in the Iberian Peninsula (Online Resource 1). Tissue samples consisted of a portion of tail (< 1 mm) collected from alive (non-euthanized) and road-killed specimens encountered during fieldwork. Capture and handling of vipers for data collection was carried out following all ethical procedures (see Ethics approval).

DNA extractions were performed using EasySpin Kit, following the manufacturer’s instructions. DNA integrity and purity were assessed by agarose gel electrophoresis (2%) and spectrophotometry. Enriched microsatellite libraries were then generated from genomic DNA at a 50 ng µL − 1 concentration using a modified protocol from [20]. Sequencing was carried out using MiSeq V2 kit (500 cycles) and single Illumina indexes.

Reads from sequencing were analysed using Micro-Primers [21] to find microsatellites using optimized parameters to detect those more polymorphic. The analysis included quality trimming using Trimmomatic, merge of pairs using Flash and microsatellites finding through MISA. The obtained microsatellites were then screened and selected to design multiplexes for each species using Multiplex Manager 1.2. The following parameters were applied for markers selection: (1) to maximize the space between markers in the same dye, (2) minimize the difference in annealing temperatures of markers in the same reaction and (3) minimize complementarity of primers in the same reaction.

Using these criteria, we selected 70 potential markers (ca. 20 for each species) that were multiplexed in two reactions for each species. See Table 1 for primer details. For each marker, a third primer was used, following the M13 tailed primer method [22]. This primer was labelled with FAM, NED, VIC or PET, depending on the selected dye for each locus (Table 1). Forward primers had a 5’ tail complementary to the fluorescently labelled primers.

**Table 1:** Characteristics of 48 polymorphic microsatellite loci developed for *V. aspis*, *V. latastei* and *V. seoanei*. *TD*: touchdown temperature of annealing

Loci	Primer (Fw)	Primer (Rv)	Repeat motif	Allele size range	Multiplex	TD	Dye
Vasp1	TTCTGCAGTTCTGGTGGTTG	AACAAGCAGTTGCTGAGCTG	(TG)15	[109,129]	1	62/58	FAM
Vasp2	CAGGCAGCCAAACTACTATGC	CTGCCTGCCTATGTCTCTCC	(GATA)23	[190,226]	1	62/58	FAM
Vasp3	AACTTGACCGCAAACTGAGC	ATTCAGACCCTTGGCAACTG	(CA)13	[256,276]	1	62/58	FAM
Vasp4	GGGCATTAGACCATGTTTGG	CAGGCCTTAGTTTATGCAGGAC	(AC)9	[107,127]	1	62/58	VIC
Vasp5	TGGGTGACACTTGACAGAGG	CACCCAAATAGGTTAGCCAGAC	(TG)19	[152,170]	1	62/58	VIC
Vasp7	TTTGTTAAGGAGGGAAAGCAAG	TTAAAGCTGCCCAAAGTTCC	(GT)19	[119,129]	1	62/58	NED
Vasp8	GATATGGAGTGGGCAGATGG	TGTGTTCAAACCTTCACATTCTG	(GT)20	[173,195]	1	62/58	NED
Vasp9	TGTTTCAGCCTAACCCAAGG	TTCTGGGAGGCACACTGAG	(AC)19	[242,266]	1	62/58	NED
Vasp10	TGAAAGGCAGGTGCTAGGTC	TGCTGGCTCCCTCAACTAAG	(TG)19	[129,145]	1	62/58	PET
Vasp11	TGGTAGAGGACAGAGGACAGAAG	AATTGGACGTCTTGGTGGAC	(GATA)21	[187,263]	1	62/58	PET
Vasp15	GATGTTCTCTTCCTCTCCCTCTC	AAGGAATATTGAACAGAACCAAGG	(AG)26	[143,169]	2	62/58	VIC
Vasp16	CCAACAATGACTCCAAGTGATG	CCAAGATTGATCAGAAGGTTCC	(ATGA)13	[203,243]	2	62/58	VIC
Vasp20	TGCTTGATCCACCTCAACAG	CTTCCATTGATTGGTTGGTTG	(GAAT)13	[260,288]	2	62/58	NED
Vasp21	GCAAGCCTGTGTTGATTCTG	GCAATTGTCACTCAGACAAAGG	(CA)20	[126,142]	2	62/58	PET
Vasp22	AGGACGATGGGAAGATGATG	TCAATCGCACACACACACAC	(GT)11	[174,192]	2	62/58	PET
Vasp23	CTCCTGAGACAGCCACTTGTC	CGTCATGTGCAAATCCACTC	(TTCT)25	[212,256]	2	62/58	PET
VLa1	GTTGACTCAGCCTTCCATCC	GGCTTTCACACACACACACC	(GT)6	[111,129]	3	60/56	FAM
VLa2	TGCAGGTAGCACACAGAAATG	TGGTGTAAGCCTGCAGAATG	(GGAA)21	[168,204]	3	60/56	FAM
VLa4	CAGACTGCTGGGAATAGATTAAGG	GGAATTAGCCTTGAAGCCTTG	(TG)23	[104,144]	3	60/56	VIC
VLa8	AAGAGAAGAAGGCGACAGACC	TTTGGGAGTGTTTGGTGACTC	(TCCT)15	[217,253]	3	60/56	NED
VLa11	CCAAAGAATGCAAGGTAGGC	CCTTCTCCCATTTCCTCTCC	(GA)6	[106,132]	4	60/56	FAM
VLa12	AAGGCTCATGGAATGATATTGG	AAGATTTGTCCCGTGTTTGG	(CT)19	[185,219]	4	60/56	FAM
VLa14	TGGGCATGTGCCATAAATAATAC	CCCTGCCAATTCTGTGATTC	(GAAG)18	[97,141]	4	60/56	VIC
VLa16	TGCTCAATCACCTCCATTAGG	AGATGGAGCAGAAGCGACTC	(TC)8	[235,255]	4	60/56	VIC
VLa17	ACATTACAGCGTGAAGACAAGAAC	TTGGAAAGCTAAGGAAGAGCAG	(CT)6	[106,154]	4	60/56	NED
VLa20	GGCAGGAATCAGAAACCAATAG	AATGAGAAAGACAAAGTGTGAGAGAG	(TCTT)23	[112,160]	4	60/56	PET
VLa21	GAGCAACCATTCTCAGAGAGC	TTCCTTCTAAAGCCGTCAGC	(TCTA)14	[223,255]	4	60/56	PET
Vse2	CAACAAATACCAGCCCATTTG	TTTAGCCATAGCCGTGTGTG	(AC)20	[156,178]	5	64/60	FAM
Vse4	GTTGCCATTTCCTTCTCCAG	CACTTTGAGAGCAAGCAACG	(AGAT)15	[278,306]	5	64/60	FAM
Vse5	TTACATCGTGGTGGATGCAG	AGGGATGGATAGAGGGATGG	(TATC)20	[121,157]	5	64/60	VIC
Vse7	TGGAGGATAATGGAACCTGTG	TTTCCATTTATTGCCAACCTG	(AC)22	[253,277]	5	64/60	VIC
Vse8	CCCTCTTTGCCTCTGTATGG	TAGGAGCCAGCATGTTTGTG	(AC)8	[106,146]	5	64/60	NED
Vse9	CATTTCCCATTTGGATCTGC	ACCTGGATAGCGGCTTCTTC	(TG)13	[180,206]	5	64/60	NED
Vse10	GATATGAGCCAGCCGTGTG	CGCCGTTGTAGCTTTGAATAG	(GT)9	[247,265]	5	64/60	NED
Vse11	GCTTTGCTGGTTAGAAGGTCAC	TCCAGATGTTTGGCCACTG	(CA)16	[103,131]	5	64/60	PET
Vse13	AGCTCATGCCTACTGCTTCAG	GGGAGAGATAAATTCTACTTCTGTCTG	(TAGA)26	[233,265]	5	64/60	PET
Vse14	AACTCCTCTTGAATTCCATCTCC	TTGAGCTCTTGGAGGAAAGG	(GT)18	[120,148]	6	62/58	FAM
Vse15	TGTAATGACAGCAGGAAGCAG	AGAAGTGGAAGCTGGCAATC	(GT)18	[200,224]	6	62/58	FAM
Vse16	GATGGACATCACTGTCTCATGTTAG	CACAAATGCAGCAAGAATGG	(TC)23	[261,281]	6	62/58	FAM
Vse18	TGTTCTGGCACCTGTTCTTG	TTGGAAGACCCAAAGGAGTG	(AC)18	[170,194]	6	62/58	VIC
Vse19	TTGAAGAAATCCACCCATCC	GAGCACAGCAACAAATGGAC	(GA)13	[228,248]	6	62/58	VIC
Vse20	TCCCAAGAAGCACTTTCCAG	GCTAAAGAACTGGCAGCTGTG	(GT)7	[265,291]	6	62/58	VIC
Vse21	ATCCAGGTCTCACATGCTCAC	GACCTGCAGCTCTATTCTGATTG	(ATCA)10	[90,110]	6	62/58	NED
Vse22	TCCGCATTTATGACATTTGC	AGGATGAGATGCTTGTTTAGGC	(TC)17	[152,170]	6	62/58	NED
Vse23	CAACTCTGGGAAATCCATCG	TGGTGGTATGAAACCCATGAC	(TATC)23	[236,268]	6	62/58	NED
Vse24	CCTTGACCTCGAAATGTTGC	AAAGCTGTCACTGGCTCACC	(GT)12	[100,118]	6	62/58	PET
Vse25	ACAAATGGCTGACCTCTCTCTC	TGTATTATTGAAATGCCTCTTTGC	(TATC)16	[167,203]	6	62/58	PET
Vse26	ATTGGGTCAGTTCCGAGATG	CCTACACACACACACACACACAC	(TG)11	[235,253]	6	62/58	PET

Multiplexes were tested for amplification and polymorphism using 26, 20 and 10 samples of *V. aspis*, *V. latastei* and *V. seoanei* respectively (Online Resource 1). PCRs were performed in a total volume of 10 µl with 1–2 µl template DNA, 100–150 nM of each primer, and 5 µl Multiplex PCR Master Mix (Qiagen). Touchdown PCR conditions started with an initial denaturation step of 15 min at 95 °C; first round (nine cycles) of 30 s at 95 °C, 2 min for annealing (-0.5 °C/cycle), and 1 min at 72 °C; second round (36 cycles) of 30 s at 95 °C, 1 min at annealing temperature, 30 s at 72 °C, and a final extension of 30 min at 60 °C. Multiplexes details and touchdown temperatures can be consulted in Table 1. Amplifications were performed in Biorad T100 Thermal Cyclers, and the PCR products were separated by capillary electrophoresis on an automatic sequencer *ABI3500xl Genetic Analyzer* (AB Applied Biosystems). Fragments were scored and manually checked by two independent persons using GENEMAPPER 5.0 (Applied Biosystems).

Individuals from the same species were pooled and three populations were defined for loci characterization. The existence of null alleles, allelic dropouts and stuttering were verified using MICROCHECKER v2.2.3. Tests of Hardy–Weinberg equilibrium (HWE) deviations for each locus and linkage disequilibrium (LD) for all pairs of loci were computed with ARLEQUIN 3.5. Basic population summary statistics such as the number of alleles per locus (N_A_), observed heterozygosity (H_O_) and expected heterozygosity (H_E_) were estimated with ARLEQUIN 3.5.

Moreover, the transferability of these markers was tested by cross amplification on the three vipers, using a subset of eight samples per species.

## Results and discussion

From the 70 primer pairs initially tested, 25 were discarded due to no amplification (or low amplification success), no polymorphism or unreliable allelic patterns that could cause potential scoring errors. In total we obtained 16, 11, and 21 loci that amplified reliably and were polymorphic within species, for *V. aspis*, *V. latastei* and *V. seoanei*, respectively (Tables 1 and 2). MICROCHECKER showed no evidence of allelic dropout or stuttering, but null alleles (unamplified alleles) were diagnosed across several loci that showed significant excess of homozygous (Table 2). Significant deviation of HWE towards heterozygosity deficiency was detected for several loci in the three species (Table 2). This can be attributed to presence of null alleles and also to the effect of population substructure, since the samples analysed were obtained from different localities, and thus they may not represent a panmictic population. Significant linkage disequilibrium (LD) was also observed across multiple loci, particularly in *V. seoanei* (Table 2). LD, which is the non-random association of alleles at different loci, can be induced by several factors, including selection and genetic drift [23]. The range expansion experienced by *V. seoanei* from northwestern Iberia likely led to profound bottleneck events and resulted in decreased genetic diversity eastward [16]. Allele surfing processes during range expansion events shape patterns of genetic structure through genetic drift (e.g. [24]), and thus can be associated to increased genome-wide LD, due to the random sampling of loci during bottleneck events. Therefore, the demographic history of this species offers an explanation for the high LD proportion observed.

**Table 2:** Genetic parameters of the 48 evaluated loci and transferability across the three *Vipera* species. NA means no amplification. Most polymorphic loci (higher N_A_, H_E_, H_O_) are highlighted in bold. Shaded areas correspond to loci for which cross-amplification tests were not performed for a given species

Locus	Species	n	N_A_	H_O_	H_E_	HWE	LD	Cross-amplification	N_A_ VAS	N_A_ VLA	N_A_ VSE
Vasp1	*V. aspis*	26	11	0.21	0.78	S*	S	yes		8	6
**Vasp2**	*V. aspis*	26	23	0.88	0.95	NS	S	no		NA	NA
**Vasp3**	*V. aspis*	26	6	0.58	0.65	NS	NS	yes		4	2
**Vasp4**	*V. aspis*	26	5	0.42	0.39	NS	S	yes		8	7
**Vasp5**	*V. aspis*	26	10	0.42	0.84	S*	S	no		NA	NA
**Vasp7**	*V. aspis*	26	9	0.81	0.86	NS	S	yes		10	7
**Vasp8**	*V. aspis*	26	5	0.54	0.60	S*	S	yes		7	10
**Vasp9**	*V. aspis*	26	10	0.80	0.81	NS	S	no		NA	NA
**Vasp10**	*V. aspis*	26	9	0.77	0.83	NS	S	yes		8	6
**Vasp11**	*V. aspis*	26	17	0.85	0.91	NS	S	no		NA	NA
**Vasp15**	*V. aspis*	26	10	0.42	0.83	S*	S	no		NA	NA
**Vasp16**	*V. aspis*	26	7	0.58	0.70	NS	S	yes		5	7
**Vasp20**	*V. aspis*	26	8	0.35	0.75	S*	S	yes		4	6
**Vasp21**	*V. aspis*	26	7	0.65	0.80	NS	S	yes		2	3
**Vasp22**	*V. aspis*	26	7	0.69	0.72	NS	S	no		NA	NA
**Vasp23**	*V. aspis*	26	11	0.44	0.88	S*	S	yes		8	9
VLa1	*V. latastei*	21	2	0.24	0.21	NS	NS	yes	7		3
**VLa2**	*V. latastei*	21	9	0.71	0.87	NS	S	yes	4		3
**VLa4**	*V. latastei*	21	11	0.95	0.82	NS	NS	yes	2		9
**VLa8**	*V. latastei*	21	11	0.67	0.88	NS	S	yes	5		8
VLa11	*V. latastei*	21	2	0.05	0.05	NS	S	no	NA		NA
**VLa12**	*V. latastei*	21	17	0.67	0.92	S*	S	no	NA		NA
**VLa14**	*V. latastei*	21	11	0.76	0.90	NS	NS	yes	5		8
**VLa16**	*V. latastei*	21	17	0.35	0.91	S*	S	yes	7		2
**VLa17**	*V. latastei*	21	7	0.40	0.40	NS	S	yes	4		1
**VLa20**	*V. latastei*	21	8	1.00	0.83	NS	S	yes	6		6
**VLa21**	*V. latastei*	21	7	0.86	0.80	NS	S	yes	8		5
**Vse2**	*V. seoanei*	10	6	0.56	0.85	S*	S	yes	6	3	
**Vse4**	*V. seoanei*	10	8	0.75	0.90	NS	S	yes	5	4	
Vse5	*V. seoanei*	10	6	0.22	0.83	S*	S	no	NA	NA	
Vse7	*V. seoanei*	10	4	0.14	0.67	NS	S	yes	4	4	
Vse8	*V. seoanei*	10	3	0.00	0.57	NS	S	no	NA	NA	
**Vse9**	*V. seoanei*	10	7	0.75	0.79	S*	S	yes	4	4	
Vse10	*V. seoanei*	10	3	0.25	0.61	S*	S	yes	4	3	
Vse11	*V. seoanei*	10	3	0.30	0.42	S*	S	yes	5	5	
**Vse13**	*V. seoanei*	10	9	0.63	0.93	NS	S	yes	4	3	
**Vse14**	*V. seoanei*	10	5	0.60	0.70	NS	S	yes	1	1	
Vse15	*V. seoanei*	10	3	0.30	0.28	NS	S	yes	7	7	
**Vse16**	*V. seoanei*	10	6	0.63	0.81	NS	S	Only in *V. aspis*	6	NA	
**Vse18**	*V. seoanei*	10	4	0.50	0.56	S*	S	Only in *V. aspis*	7	NA	
**Vse19**	*V. seoanei*	10	5	0.80	0.77	NS	S	yes	5	6	
Vse20	*V. seoanei*	10	3	0.25	0.34	NS	S	no	NA	NA	
**Vse21**	*V. seoanei*	10	5	0.50	0.79	NS	S	yes	4	5	
Vse22	*V. seoanei*	10	2	0.40	0.34	NS	S	no	NA	NA	
**Vse23**	*V. seoanei*	10	5	0.75	0.81	S*	S	no	NA	NA	
Vse24	*V. seoanei*	10	3	0.50	0.43	NS	S	yes	10	7	
**Vse25**	*V. seoanei*	10	6	0.70	0.77	NS	S	yes	3	4	
**Vse26**	*V. seoanei*	10	5	0.86	0.79	NS	S	yes	3	3	

n: sample size of each population. N_A_: number of alleles. H_O_, H_E_: observed and expected heterozygosity. HWE: Hardy-Weinberg equilibrium test (NS means not significant/ S means significant, *p* value = 0.05). * in Hardy-Weinberg equilibrium tests means that the presence of null alleles is suspected. LD: Linkage disequilibrium test (NS means not significant/ S means significant, *p* value = 0.05). VAS: *V. aspis*, VLA: *V. latastei*, VSE: *V. seoanei*

For *V. aspis*, the number of alleles (N_A_) ranged between 5 (Vasp8) to 23 (Vasp2), with a mean number of alleles per locus of 10. The majority of loci had moderate to high observed (minimum of 0.21 in Vasp1 to 0.88 in Vasp2) and expected heterozygosity (H_E_ = 0.40 in Vasp4, 0.95 in Vasp2). For *V. latastei*, N_A_ ranged between two (VLa1 and VLa11) to 17 (VLa12 and VLa16), with a mean number of alleles per locus of nine. Observed and expected heterozygosity varied between locus from very low to high (H_O_ = 0.05 in VLa11 and 1.0 in VLa20; H_E_ = 0.05 in VLa11 and 0.92 in VLa12). For *V. seoanei*, number of alleles per locus ranged from 2 (Vse22) to 9 (Vse13) with a mean of 5. Observed and expected heterozygosity also ranged from very low to high (H_O_ = 0.05 in Vse8, and 0.86 in Vse26; H_E_ = 0.28 in Vse15, and 0.93 in Vse13). Out of the 48 loci analysed, 36 are highly polymorphic (high N_A_, H_E_, H_O_) and have the potential to be informative in future studies that encompasses other populations and subspecies (Table 2).

Cross-species transferability was achieved for 31 microsatellites loci that consistently amplified and were polymorphic in the three target species and four additional loci that were transferable to only one of the species (Table 2). Cross-amplification failed for six *V. aspis* (Vasp 2, 5, 9, 11, 15 and 22), five *V. seoanei* (Vse 5, 8, 20, 22, and 23) and two *V. latastei* markers (VLa 11 and 12). Two *V. seoanei* markers (Vse 16 and 18) failed to amplify in *V. latastei* only. Additionally, VLa17 and Vse14 were monomorphic for at least one of the species. The cross-species amplification efficiency is usually inversely proportional to the phylogenetic distance between the species [25]. However, we did not observe a lower amplification success in *V. seoanei* compared to the closely-related *V. aspis* and *V. latastei*. In fact, *V. seoanei* markers showed high applicability potential in the other two species, and vice-versa, supporting a broader use of the set of loci generated in this study in other *Vipera* species.

## Conclusion

Our study provides a selection of highly polymorphic microsatellite markers that can deepen current knowledge on genetic patterns, demographic dynamics, systematics, reproduction and heritability in these species, and potentially in other *Vipera* taxa.

## Electronic supplementary material

Below is the link to the electronic supplementary material.

Supplementary Material 1

## Data Availability

Data generated during the current study is available on request.
