# Supplementary material for: Isolation and characterization of polymorphic microsatellite loci for the three Iberian vipers, Vipera aspis, V. latastei and V. seoanei by Illumina MiSeq sequencing
Source: Mol Biol Rep. 2024 Feb 9;51(1):294. doi: 10.1007/s11033-024-09263-5 (PMC10857953; doi:10.1007/s11033-024-09263-5)
Supplement: Supplementary file 1 — Supplementary Material 1 [file 11033_2024_9263_MOESM1_ESM.pdf]

ONLINE RESOURCE 1

Title: Isolation and characterization of polymorphic microsatellite loci for the three Iberian vipers, *Vipera aspis*, *V. latastei* and *V. seoanei* by Illumina MiSeq sequencing

Journal: Molecular Biology Reports

Authors: Inês Freitas[1,2,3]*, Guillermo Velo-Antón[1,4], Susana Lopes[1,2], Antonio Muñoz-Merida[1,2], Fernando Martínez-Freiría[1,2]

Affiliations: 1. CIBIO, Centro de Investigação em Biodiversidade e Recursos Genéticos, InBIO Laboratório Associado, Campus de Vairão, Universidade do Porto, 4485-661 Vairão, Portugal; 2. BIOPOLIS Program in Genomics, Biodiversity and Land Planning, CIBIO, Campus de Vairão, 4485-661 Vairão, Portugal; 3. Departamento de Biologia, Faculdade de Ciências, Universidade do Porto, 4099-002 Porto, Portugal; 4. Universidad de Vigo, Facultad de Biología, Edificio de Ciencias Experimentales, Bloque B, Planta 2, Laboratorio 39 (Grupo GEA), E-36310, Vigo, Spain

*corresponding author (ifinesfreitas92@gmail.com)

Online Resource 1. Sampling localities used for population genetic analyses and for genomic libraries preparation (highlighted in grey).

Samples	Species	Locality	Region	Country	Latitude	Longitude
15VA115	*Vipera aspis*	Villagalijo	Burgos	Spain	42.35	-3.18
15VA122	*Vipera aspis*	Cañas	La Rioja	Spain	42.39	-2.87
15VA123	*Vipera aspis*	Cañas	La Rioja	Spain	42.39	-2.87
15VA171	*Vipera aspis*	Ezcaray	La Rioja	Spain	42.24	-3.03
15VA176	*Vipera aspis*	Ojacastro	La Rioja	Spain	42.35	-2.98
15VA188	*Vipera aspis*	Pazuengos	La Rioja	Spain	42.28	-2.95
15VA189	*Vipera aspis*	Pazuengos	La Rioja	Spain	42.28	-2.95
15VA190	*Vipera aspis*	Pazuengos	La Rioja	Spain	42.28	-2.95
15VA191	*Vipera aspis*	Pazuengos	La Rioja	Spain	42.28	-2.95
15VA192	*Vipera aspis*	Pazuengos	La Rioja	Spain	42.28	-2.95
15VA199	*Vipera aspis*	Cañas	La Rioja	Spain	42.39	-2.87
16VA058	*Vipera aspis*	Cañas	La Rioja	Spain	42.39	-2.87
16VA059	*Vipera aspis*	Cañas	La Rioja	Spain	42.39	-2.87
16VA061	*Vipera aspis*	Cañas	La Rioja	Spain	42.39	-2.87
16VA116	*Vipera aspis*	Ezcaray	La Rioja	Spain	42.30	-2.90
17VA111	*Vipera aspis*	Ezcaray	La Rioja	Spain	42.22	-3.06

17VA139	*Vipera aspis*	Santurdejo	La Rioja	Spain	42.39	-2.97
17VA149	*Vipera aspis*	San Asensio de los Cantos	La Rioja	Spain	42.37	-2.99
17VA172	*Vipera aspis*	Ojacastro	La Rioja	Spain	42.34	-3.01
18VA154	*Vipera aspis*	Ezcaray	La Rioja	Spain	42.31	-2.96
18VA155	*Vipera aspis*	Ezcaray	La Rioja	Spain	42.31	-2.96
18VS076	*Vipera seoanei*	Bricia	Burgos	Spain	42.90	-3.83
18VS077	*Vipera seoanei*	Bricia	Burgos	Spain	42.90	-3.83
19VA142	*Vipera aspis*	Ezcaray	La Rioja	Spain	42.26	-2.97
19VA240	*Vipera aspis*	Villarejo	La Rioja	Spain	42.38	-2.88
19VA462	*Vipera aspis*	Fresneda de la Sierra Tirón	Burgos	Spain	42.27	-3.14
19VL172	*Vipera latastei*	Mozuelos de Sedano	Burgos	Spain	42.69	-3.76
19VL211	*Vipera latastei*	Mozuelos de Sedano	Burgos	Spain	42.69	-3.75
20VA108	*Vipera aspis*	Ezcaray	La Rioja	Spain	42.29	-3.06
20VA194	*Vipera aspis*	Grañon	La Rioja	Spain	42.46	-3.03
20VS130	*Vipera seoanei*	Renedo de Bricia	Burgos	Spain	42.92	-3.84
20VS131	*Vipera seoanei*	Renedo de Bricia	Burgos	Spain	42.92	-3.84
20VS132	*Vipera seoanei*	Villanueva-Carrales	Burgos	Spain	42.93	-3.83
4VL028	*Vipera latastei*	Sedano	Burgos	Spain	42.72	-3.74
4VL040	*Vipera latastei*	Sedano	Burgos	Spain	42.70	-3.73
4VL054	*Vipera latastei*	Sedano	Burgos	Spain	42.72	-3.72
4VL056	*Vipera latastei*	Sedano	Burgos	Spain	42.71	-3.71
4VL116	*Vipera latastei*	Quintanaloma	Burgos	Spain	42.70	-3.68
4VL144	*Vipera latastei*	Sedano	Burgos	Spain	42.70	-3.73
4VL149	*Vipera latastei*	Sedano	Burgos	Spain	42.70	-3.73
4VL162	*Vipera latastei*	Mozuelos de Sedano	Burgos	Spain	42.67	-3.74
5VL025	*Vipera latastei*	Sedano	Burgos	Spain	42.71	-3.75
5VL026	*Vipera latastei*	Mozuelos de Sedano	Burgos	Spain	42.67	-3.75
5VL030	*Vipera latastei*	Covanera	Burgos	Spain	42.74	-3.80
5VL038	*Vipera latastei*	Sedano	Burgos	Spain	42.69	-3.73
5VL052	*Vipera latastei*	Sedano	Burgos	Spain	42.71	-3.73
5VL074	*Vipera latastei*	Sedano	Burgos	Spain	42.71	-3.73
5VL075	*Vipera latastei*	Sedano	Burgos	Spain	42.66	-3.75
5VL117	*Vipera latastei*	Mozuelos de Sedano	Burgos	Spain	42.66	-3.75
5VL156	*Vipera latastei*	Sedano	Burgos	Spain	42.70	-3.73
5VL202	*Vipera latastei*	Villagalijo	Burgos	Spain	42.66	-3.75
5VS065	*Vipera seoanei*	Valderredible	Cantabria	Spain	42.85	-3.91
5VS069	*Vipera seoanei*	Sargentes de la Lora	Burgos	Spain	42.77	-3.94
5VS070	*Vipera seoanei*	Sargentes de la Lora	Burgos	Spain	42.77	-3.93
5VS143	*Vipera seoanei*	Valderredible	Cantabria	Spain	42.79	-3.93
5VS154	*Vipera seoanei*	Valderredible	Cantabria	Spain	42.84	-3.88
10VS001	*Vipera seoanei*		Galicia	Spain	41.91	-7.93
12VS002	*Vipera seoanei*		Galicia	Spain	42.26	-7.36
12VS003	*Vipera seoanei*		Galicia	Spain	42.65	-7.33
12VS004	*Vipera seoanei*		Cantabria	Spain	43.39	-4.43
12VS010	*Vipera seoanei*		León	Spain	42.89	-6.20
12VS014	*Vipera seoanei*		Cantabria	Spain	43.13	-3.73
12VS023	*Vipera seoanei*		Cantabria	Spain	43.37	-3.20
VSCB005	*Vipera seoanei*		Viana do Castelo	Portugal	41.87	-8.51

10VS013	*Vipera seoanei*	Viana do Castelo	Portugal	42.07	-8.14
17VL192	*Vipera latastei*	Galicia	Spain	42.12	-7.15
P166	*Vipera latastei*	Beira Alta	Portugal	40.40	-7.51
P158	*Vipera latastei*	Algarve	Portugal	37.30	-8.57
15VL015	*Vipera latastei*	Aragón	Spain	42.16	0.47
P212	*Vipera latastei*	Andalucía	Spain	38.21	-3.95
15VL013	*Vipera latastei*	Murcia	Spain	37.86	-1.54
13VL027	*Vipera latastei*	Castilla La Mancha	Spain	40.40	-1.97
15VL079	*Vipera latastei*	Andalucía	Spain	36.26	-5.48
6VA060	*Vipera aspis*	Burgos	Spain	42.73	-3.79
15VA017	*Vipera aspis*	Castilla y León	Spain	42.64	-3.10
15VA022	*Vipera aspis*	La Rioja	Spain	42.22	-2.28
15VA080	*Vipera aspis*	Navarra	Spain	42.95	-1.29
15VA090	*Vipera aspis*	Pyrennes	France	42.86	-0.39
15VA092	*Vipera aspis*	Pyrennes	France	42.92	0.35
15VA103	*Vipera aspis*	Euskadi	Spain	42.63	-2.68
15VA104	*Vipera aspis*	Catalunya	Spain	42.59	0.99
15VA107	*Vipera aspis*	Aragón	Spain	42.77	-0.52
